# Temporal evolution of PM_2.5_, PM_10_, and total suspended particles (TSP) in the Ciuc basin (Transylvania) with specific microclimate condition from 2010 to 2019

**DOI:** 10.1007/s10661-023-11407-2

**Published:** 2023-06-02

**Authors:** Katalin Bodor, Róbert Szép, Ágnes Keresztesi, Zsolt Bodor

**Affiliations:** 1 Faculty of Economics, Department of Bioengineering, Sapientia Hungarian University of Transylvania, Socio-Human Sciences and Engineering, Libertății Sq. 1, 530104 Miercurea Ciuc, Romania; 2 Faculty of Natural Sciences, Doctoral School of Chemistry, University of Pécs, St. Ifjúság 6, 7624 Pécs, Hungary; 3 Institute for Research and Development of Hunting and Mountain Resources, St. Progresului 35B, 530240 Miercurea Ciuc, Romania

**Keywords:** Air pollutants, Particulate matter, PM_2.5_, PM_10_, TSP

## Abstract

Modern societies are characterized by increased air pollution, and particulate matter (PM) is one of the most significant air pollutants and is a major environmental health problem. Therefore, long- and short-term exposure via inhalation, ingestion, and dermal absorption of particulate matter may cause series health issues, such as cardio pulmonary and lung cancer disease. Air pollutants accumulation is significantly higher in closed regions or basins characterized by strong thermal inversions, especially during the cold period such in case of the Ciuc basin. The aim of this study was to carry out the time series analysis of PM_2.5_, PM_10_ and TSP in the Ciuc basin for the period 2010–2019, in order to decipher the main characteristics of air pollution in this region.

The data obtained were analyzed on a daily, monthly and annual basis by different statistical methods. The average monthly concentration of TSP (60.03 µg m^−3^), PM_10_ (19.21 µg m^−3^) and PM_2.5_ (14.73 µg m^−3^) particulate matter in the studied regions varied between 29.84–134.79 µg m^−3^, 4.38–63.51 µg m^−3^ and 4.01–54.41 µg m^−3^, respectively. Regarding the ratio of PM_2.5_ and PM_10_ in the total particulate matter (TPM) was 0.25 and 0.33. Due to meteorological factors and emission fluctuations, particulate matter exhibits high seasonal variations, therefore the highest concentrations were recorded during the cold period, while the lowest values were observed in summer. The percentage of PM_10_ exceedances (50 µg m^−3^) represents 24.8% in winter, meanwhile in autumn and spring a significantly lower exceedances percentage was observed, 2.6% and 1.7%, respectively. The correlation analysis revealed that the correlation level of the studied pollutants varied between 0.73–0.78.

## Introduction

Globally, air pollution has become one of the most severe issues. A significant number of premature deaths, increased morbidity, and economic losses are caused by particulate matter (PM) present in the air. On the other hand, population growth and economic development have resulted in a large number of air pollutants, namely particulate matter and other toxic elements and gases as well. PM interacts with the environment and there are three ways in which particles can enter into the human body: by ingestion, inhalation and dermal absorption (K. Bodor et al., 2021a, 2021b; Bodor et al., 2021b; EPA, 2004).

The particulate matter definition generally refers to a mixture of solid and liquid particles suspended in air (Johnston et al., 2019). Regarding the source of major air pollutants, two main groups can be distinguished: air pollutants from natural (forest fires, volcanic eruptions, etc.) and anthropogenic (from traffic, industrial emissions, etc.) sources. According to the reports of the World Health Organization (WHO), air pollution represents one of the biggest environmental risks for human health, 22% of illnesses and deaths can be linked to air pollution (Prüss-Üstün et al., 2016). Particles found in the atmosphere can come from two different sources—primary and secondary. Primary particles are emitted directly into the atmosphere while secondary particles are coming from the chemical reaction of gases (EPA, 2003). The particulate matter aerodynamic diameter is very important parameter because it can provide valuable information about the source and possible deposition of PM particles in different tissues and organs in the human body (Liu et al., 2016; Vlachogiannis et al., 2021). The aerodynamic diameter of PM’s is in the range of 0.001 to 500 μm, therefore PM, including the PM_10_ (coarse particulate) and PM_2.5_ (fine particulate), has wide-ranging deleterious effects on human health, especially PM_2.5_ can enter via the respiratory system, and the accumulation over time can result in a variety of inflammatory responses (Moran-Zuloaga et al., 2021; Sharma et al., 2019).

Several epidemiological studies suggest that particulate matter concentrations negatively affect the quality of life and can cause a variety of health problems, such as the deterioration of lung function, which is the result of increased use of medication (Consonni et al., 2018; Franklin et al., 2015; Künzli et al., 2000; Vlachogiannis et al., 2021; Zhang & Tripathi, 2018). Short-term symptoms of exposure to air pollution include itchy eyes, sore nose and throat, wheezing, coughing, shortness of breath, chest pain, headache, nausea, and respiratory infections (Bhatti et al., 2021). Long-term exposure to high concentrations of PM_10_ can cause a range of health effects and premature death, such as lung cancer, cardiovascular disease, chronic respiratory disease and allergies as well (Consonni et al., 2018; Pozza et al., 2010; Xia et al., 2016; Yan et al., 2019).

## Materials and Methods

### Study area location

**Fig. 1:**
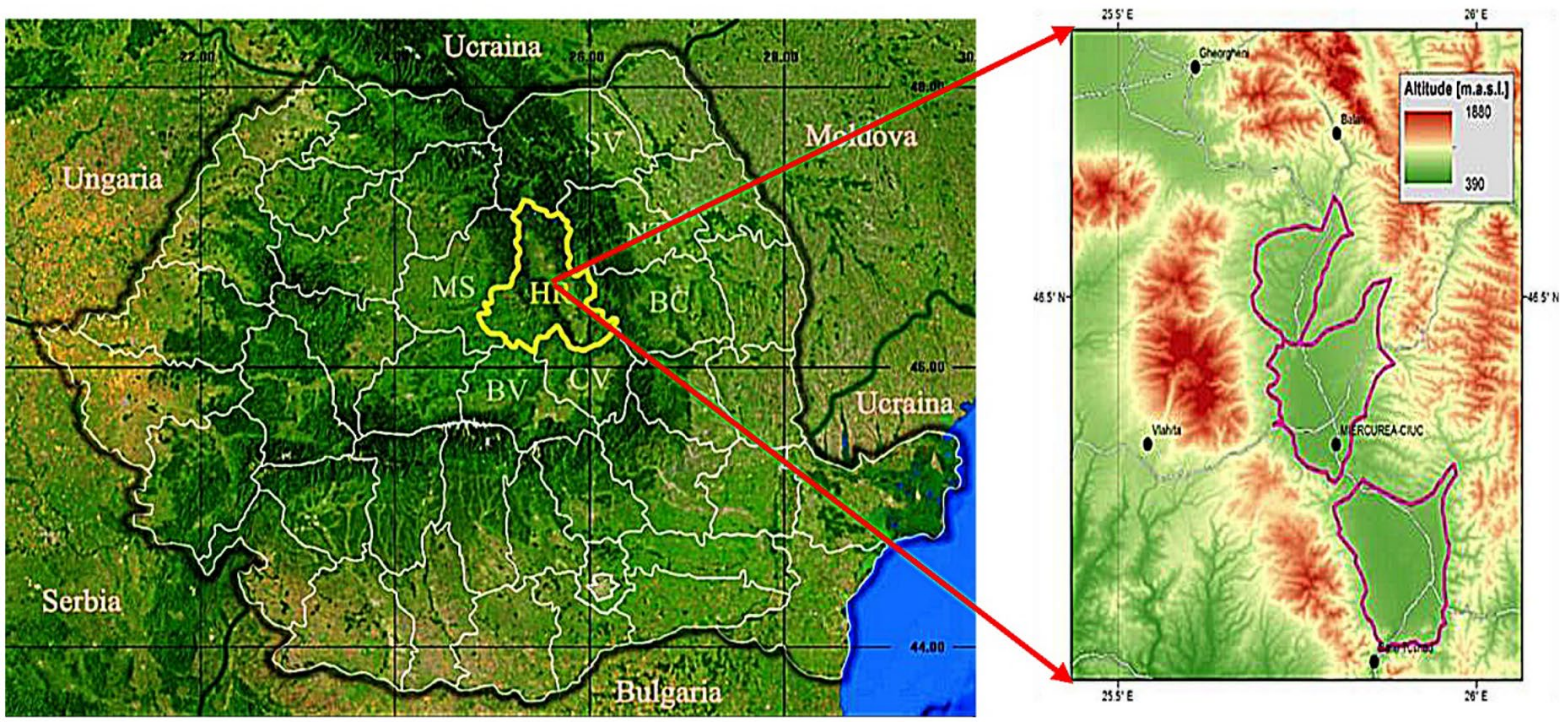
Ciuc basin sampling site geolocation (Bodor et al. 2021b; “https://www.wikiwand.com/ro/Jude%C8%9Bul_Harghita” n.d)

The sampling site—Ciuc basin—is an intra-mountain basin at an altitude of 600–700 m in the Eastern Carpathians, Harghita County, Romania (Fig. 1). It is located between the Harghita Mountains and the Ciuc Mountains, and it runs north south and is 60 km long, 10 km wide at its widest point, and is crossed by the Olt River. It is divided into three distinct geographical regions: Upper lane, Middle lane and Lower lane.

The Ciuc basin has a specific microclimate as a result of geographical and climate conditions (closed basin), with long episodes of atmospheric stability and thermal inversion periods, especially during winter, hence favoring the accumulation of pollutants (Bodor et al., 2021b). According to this phenomena a clear seasonal pattern can be observed in the case of major air pollutants concentrations (Bodor et al., 2021b; Z. Bodor et al., 2020).

### Air pollution data and meteorological parameters

The air pollution data (TSP, PM_10_, PM_2.5_) and meteorological parameters (air temperature, relative humidity, precipitation quantity, air pressure and solar radiation) were obtained from the National Air Quality Monitoring Network, where the concentrations of TSP, PM_10_ and PM_2.5_ were measured by the HR_01_ regional monitoring station located near the municipality of Miercurea Ciuc, Romania, at 46.33°N, 25.81°E, at an elevation of 697 m a.s.l. The air quality raw data covering the period 2010–2019 was collected, using daily mean particulate matter concentrations determined by gravimetric methods: EN12341.

### Statistical analysis

In order to determine the monthly, seasonal and yearly TSP, PM_10_ and PM_2.5_ concentrations, the daily gravimetric measurements were used in statistical analyses. The particulate matter concentration was determined using different statistical indices: mean, standard deviation, coefficient of variation, minimum, median, maximum, 25^th^ and 75^th^ percentile values, and confidence interval. For the normality test the Kolmogorov–Smirnov test was used in IBM SPSS 22, statistics program. Spearman correlation coefficient were calculated between the studied parameters using the R 4.2.2 statistical program. The seasonal variation of particulate matter was carried out separately taking into consideration the four different seasons (spring, summer, autumn and winter). The level of PM pollution was grouped into four categories according to Table 1.

**Table 1:** Different pollution levels according to particulate matter concentrations

PM types	Low	Moderate	High	Very high
Unit	µg m^−3^	µg m^−3^	µg m^−3^	µg m^−3^
TSP	< 50	50–75	75–100	> 100
PM_10_	< 25	25–37.5	37.5–50	> 50
PM_2.5_	< 12.5	12.5–18.75	18.75–25	> 25

Finally, correlation analysis was carried out using the IBM SPS statistical program and the correlations are presented in a simple scatter plot.

## Results and Discussion

### Descriptive Statistics

Firstly, the data collected were evaluated by using descriptive statistics and the results are presented in Table 2, and the baseline concentration was calculated using the 25 percentile. According to the results, the baseline concentration of the studied parameters was 38.93 μg m^−3^ (TSP), 10.77 μg m^−3^ (PM_10_) and 6.37 μg m^−3^ (PM_2.5_), respectively. On average the multiannual concentration was 60.03 μg m^−3^ for TSP, 19.21 μg m^−3^ for PM_10_ and 14.73 μg m^−3^ for PM_2.5_. Due to the seasonal variation of the emissions of different types of pollutant and meteorological parameters the air pollution level showed a clear seasonal pattern, namely significantly higher concentrations were recorded during the cold period. In case of the TSP the minimum and maximum concentration was 29.84 μg m^−3^ and 134.79 μg m^−3^, respectively. On the other hand, the minimum and maximum PM_10_/PM_2.5_ concentrations were between 4.38 μg m^−3^/4.01 μg m^−3^ and 63.51 μg m^−3^/54.41 μg m^−3^.

**Table 2:** Summary statistics of the studied air pollution

	TSP	PM_10_	PM_2.5_	Precip. *	Pres	Rad	Temp	RH
	µg m^−3^	µg m^−3^	µg m^−3^	mm	mbar	W/m^2^	°C	%
Avg	60.03	19.21	14.73	2.32	931	879	-4.7	87
stdev	24.74	13.04	13.4	3.14	4	155	2.2	6
min	29.84	4.38	4.01	0.05	926	655	-9.8	76
25P	38.93	10.77	6.37	0.62	929	774	-5.7	83
Median	53.44	14.36	9.87	0.93	930	892	-3.9	86
75P	73.63	19.72	14.73	1.95	933	987	-3.3	91
max	134.79	63.51	54.41	9.23	937	1143	-2.4	98
CV	0.41	0.67	0.9	1.35	0.00	0.18	-0.48	0.07
Period	2010–2019		2017–2019	2010–2019				

Where, ***Avg***—average, ***stdv***—standard deviation, ***min***—minimum, ***25P***—25% percentile, ***75P***—75% percentile, ***max***—maximum, ***CV***—Coefficient of variation, * daily data

In order to decipher the characteristics of data distribution the normality test was carried out, using the Kolmogorov–Smirnov test in SPSS, and according to the results, the data are non-normally distributed (Table 3).

**Table 3:** Test results of normality tests

Tests of Normality						
	Kolmogorov-Smirnov^a^			Shapiro–Wilk		
	Statistic	df	Sig	Statistic	df	Sig
TSP	0.166	116	0.000	0.903	116	0.000
PM_10_	0.240	116	0.000	0.781	116	0.000
PM_2.5_	0.250	23	0.001	0.731	23	0.000
Precipitation	0.243	122	0.000	0.589	122	0.000
Pressure	0.179	122	0.000	0.670	122	0.000
Radiation	0.133	122	0.000	0.921	122	0.000
Temperature	0.113	122	0.001	0.938	122	0.000
Relative humidity	0.055	122	0.200^*^	0.987	122	0.275

a.Lilliefors Significance Correction

## Temporal analysis of particulate matter concentration

### Monthly mean concentration evaluation

**Fig. 2:**
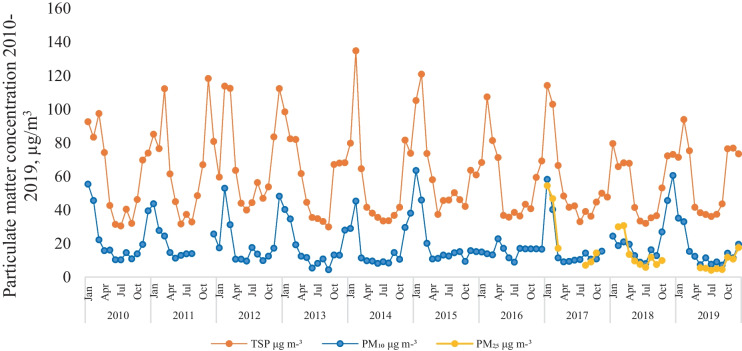
Monthly variation of particulate matter concentrations

The monthly mean concentration variations of TSP, PM_10_ and PM_2.5_ are depicted in Fig. 2, and according to the results repetitive periodic cycles and patterns can be observed. In the case of PM_2.5_ the monthly variations are presented only for the last three years of the studied period (2017–2019) due to the lack of data. The maximum concentration was detected in the winter season, from December to February, where the TSP and PM_10_ concentrations exceeded 100 μg m^−3^ and 50 μg m^−3^, values which are considered very unhealthy. As stated out earlier, the PM_2.5_ concentrations were available from 2017, and despite of that the variation pattern was similar to that observed for TSP and PM_10_, namely significantly higher concentrations during the cold period and lower levels in warmer seasons. Albeit the fact that the PM_2.5_ (10 g m^−3^) annual allowable limits are half as high as the PM_10_ (20 g m^−3^) levels, the PM_2.5_ can still have more serious health effects because of its smaller aerodynamic diameter it can penetrate the most vulnerable areas of the human organ.

### Multiannual monthly mean concentration

**Fig. 3:**
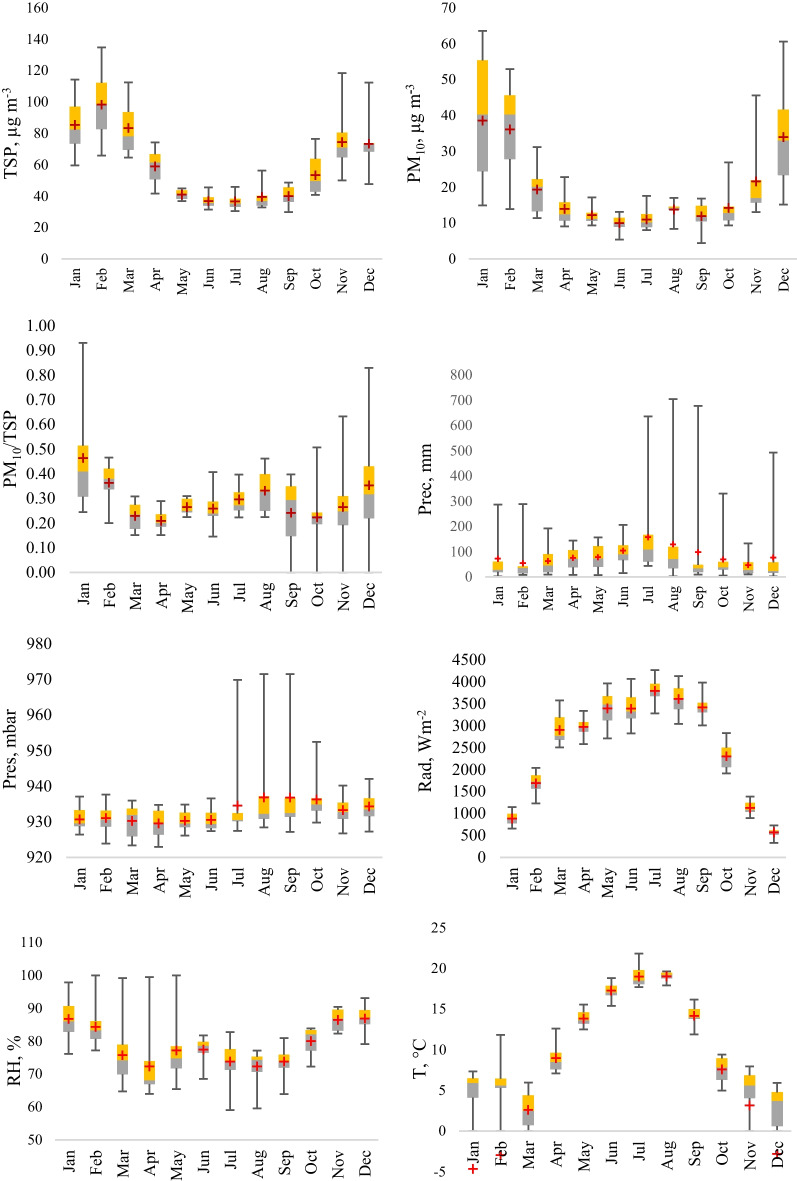
Multiannual monthly variation of particulate matter concentrations and meteorologically parameters

The multiannual monthly mean particulate matter concentrations and meteorological parameters were analyzed separately and the monthly boxplots of the results are presented in Fig. 3**.** Regarding the TSP concentration, the highest concentration (approximately 100 μg m^−3^) was measured during the cold period, which was 2.5 times higher than those values recorded in the warm period ~ 40 μg m^−3^. Furthermore, a similar tendency was observed for PM_10_, namely ~ 38 μg m^−3^ in warm periods and ~ 12 μg m^−3^ in cold periods, therefore the difference was 3 times higher between the cold and warm season. Since the PM_2.5_ concentrations were not available for the whole studied period, the data were validated only for three years (2017–1019), the multiannual monthly evaluation was not statistically representative and hence were excluded from the analyses.

### Annual mean concentration variation

**Fig. 4:**
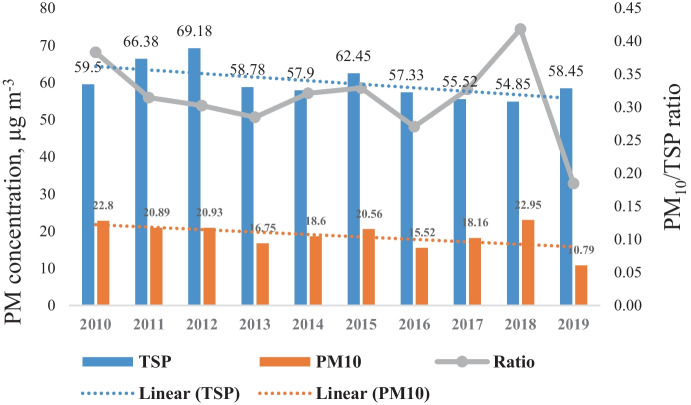
Annual variation of particulate matter concentrations

According to the results (Fig. 4) a slow decreasing tendency characterizes the evolution of TSP and PM_10_ concentrations over the studied period. Taking into account the TSP average yearly concentration evaluation, we can conclude, that the first three years (2011–2012) have shown a significant increase, with 11.51% and 16.26% increase over the first reference year (2010). Beginning from 2013 (except 2015) an average decrease of 4% was identified. Furthermore, the result revealed that the PM_10_ concentration, except for the years 2015 and 2018, shows a decreasing tendency overtime. Analyzing this tendency, the multiannual average decrease was around 20%, compared to the first reference year (2010). On the other hand, the ratio between PM_10_ and TSP on average was 0.31, however, the yearly variation ranged between 0.27 and 0.42.

### Seasonal variation of particulate matter

**Fig. 5:**
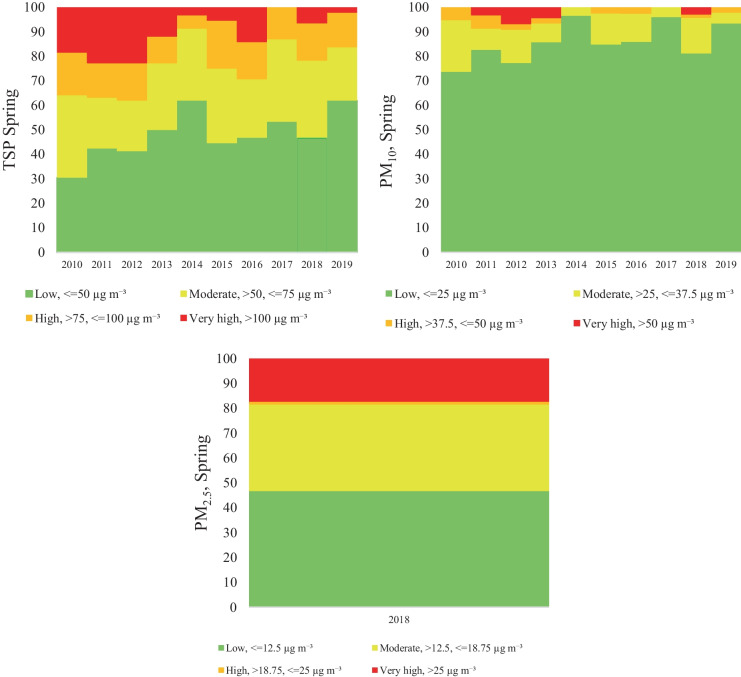
Air pollutant (TSP, PM_10_, PM_2,5_) variation in Spring

**Fig. 6:**
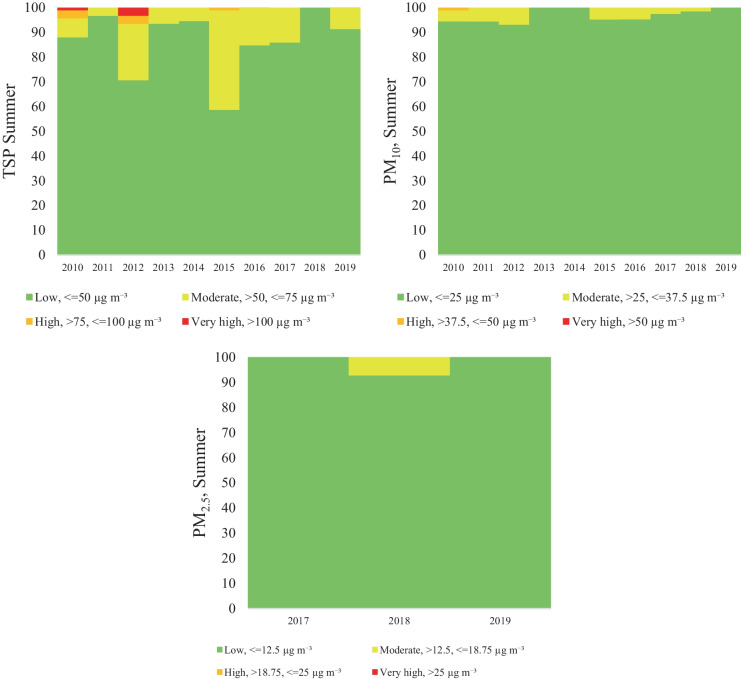
Air pollutant (TSP, PM_10_, PM_2,5_) variation in Summer

**Fig. 7:**
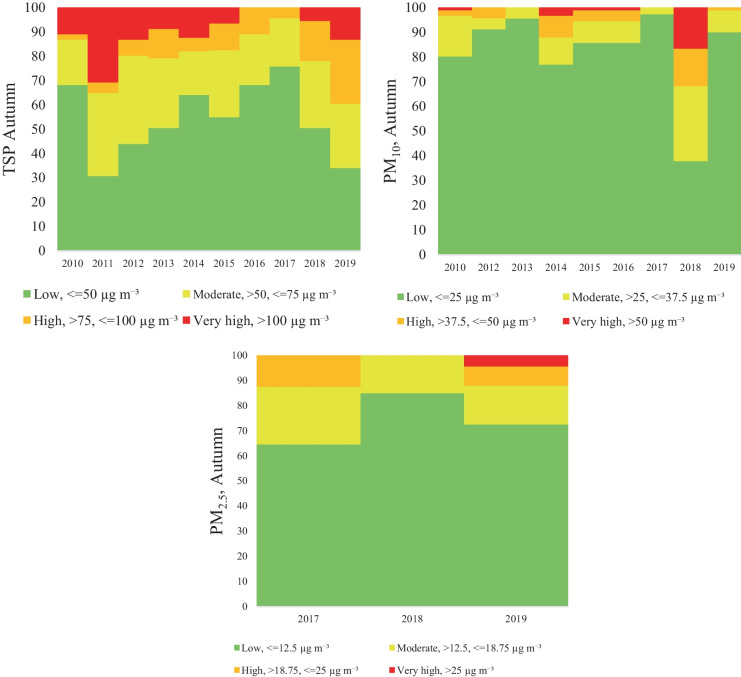
Air pollutant (TSP, PM_10_, PM_2,5_) variation in Autumn

**Fig. 8:**
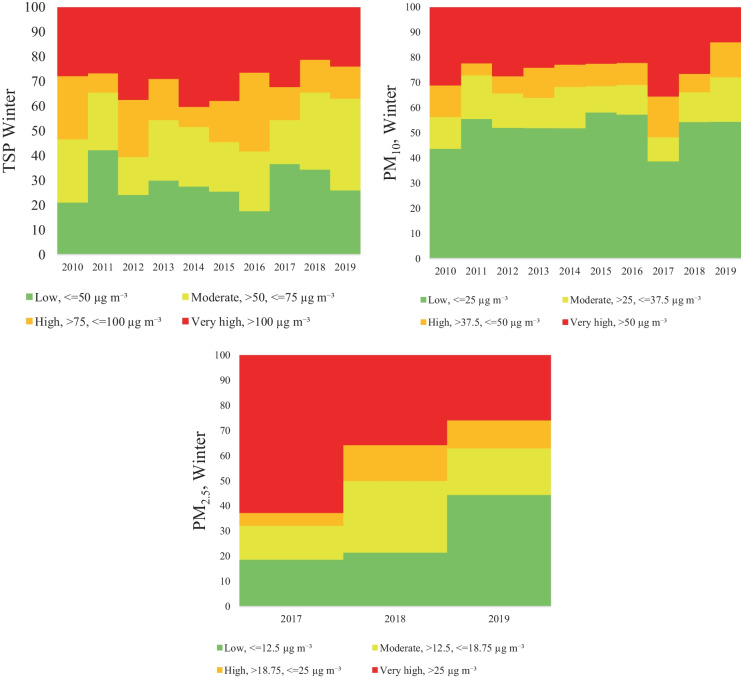
Air pollutant (TSP, PM_10_, PM_2,5_) variation in Winter

Considering the particulate matter concentration, four categories were separately established in each season. In the case of the TSP categories, it was obvious, that the highest TSP concentration was detected in the winter period, the percentage of the exceeding day (very high) on average was 30.2% (21.1–37.8%) of the total winter days. Moreover, the high TSP concentrations represent 16.9%. The moderate and low concentrations were on average 24.3% and 28.5%, respectively. The distribution of TSP concentration in autumn and spring was similar, with 10.1%/10.8% very high, 10,0%/14% high, 25.8%/27.3% moderate and 54.1%/47.9% low concentration. The lowest concentration was observed is summer with 0.4% very high, 0.8% high, 12.4% moderate and 86.4% low concentration (Figs. 5, 6, 7, and 8).

Furthermore, according to expectations, the seasonal variation of the PM_10_ concentration shows a similar trend, low concentration in summer (0% very high concentration, 0.1% high concentration, 3% moderate, and 96.8% low concentration) and high concentration in winter (24.8% very high concentration, 10% high concentration, 13.4% moderate, and 51.8% low concentration). In transitional seasons, in autumn and spring, the concentration distribution follows a similar pattern: 2.6%/1.7% very high, 4.5%/2.4% high, 10.6%/10.1% moderate and 82.3%/85.7% low concentration (Figs. 5, 6, 7, and 8).

Regarding PM_2.5_ the seasonal categories were calculated only for the last 3 years (2017–2019), since no data were available for the entire period. The results revealed higher concentrations in winter and spring, and the seasonal categories for winter are distributed as follows: 43.8% very high concentration, 10.2% high concentration, 20.2% moderate and 28.2% low concentration. In case of spring the category distribution was the following: 17.4% very high concentration, 1.1% high concentration, 34.8% moderate and 46.7% low concentration. On the other hand, in autumn the concentration had a different variation, compared to winter or spring, therefore the following concentration distributions were found: 1.5% very high concentration, 6.7% high concentration, 17.8% moderate, and 74% low concentration. Finally, in the summer period the very high and the high categories were not present (Figs. 5, 6, 7, and 8).

### *Correlation analysis of TSP, PM*_*10*_* and PM*_*2.5*_

**Fig. 9:**
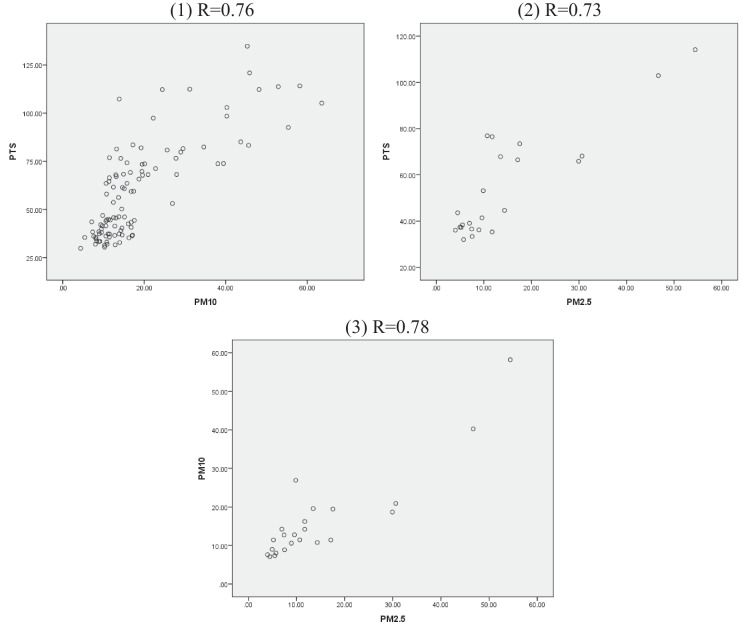
Correlations and coefficients of the studied pollutants

The inter-correlations between the studied pollutants (PM_2.5_, PM_10_, and TSP) were calculated and the results are presented in Fig. 9. The first scatter plot represents the correlation between PM_10_ (x-axis) and TSP (y-axis). The second plot shows the correlation between PM_2.5_, (x-axis) and TSP (y-axis), while the final plot represents the correlation between PM_2.5_, (x-axis) and PM_10_ (y-axis). According to the results, the highest correlation was found between PM_2.5_ and PM_10_ which represents a strong relation (r = 0.78). Moreover, the relationship between PM_2.5_ and TSP and PM_10_ and TSP was also found to be high, with correlation coefficients of r = 0.73 and r = 0.76, respectively.

**Fig. 10:**
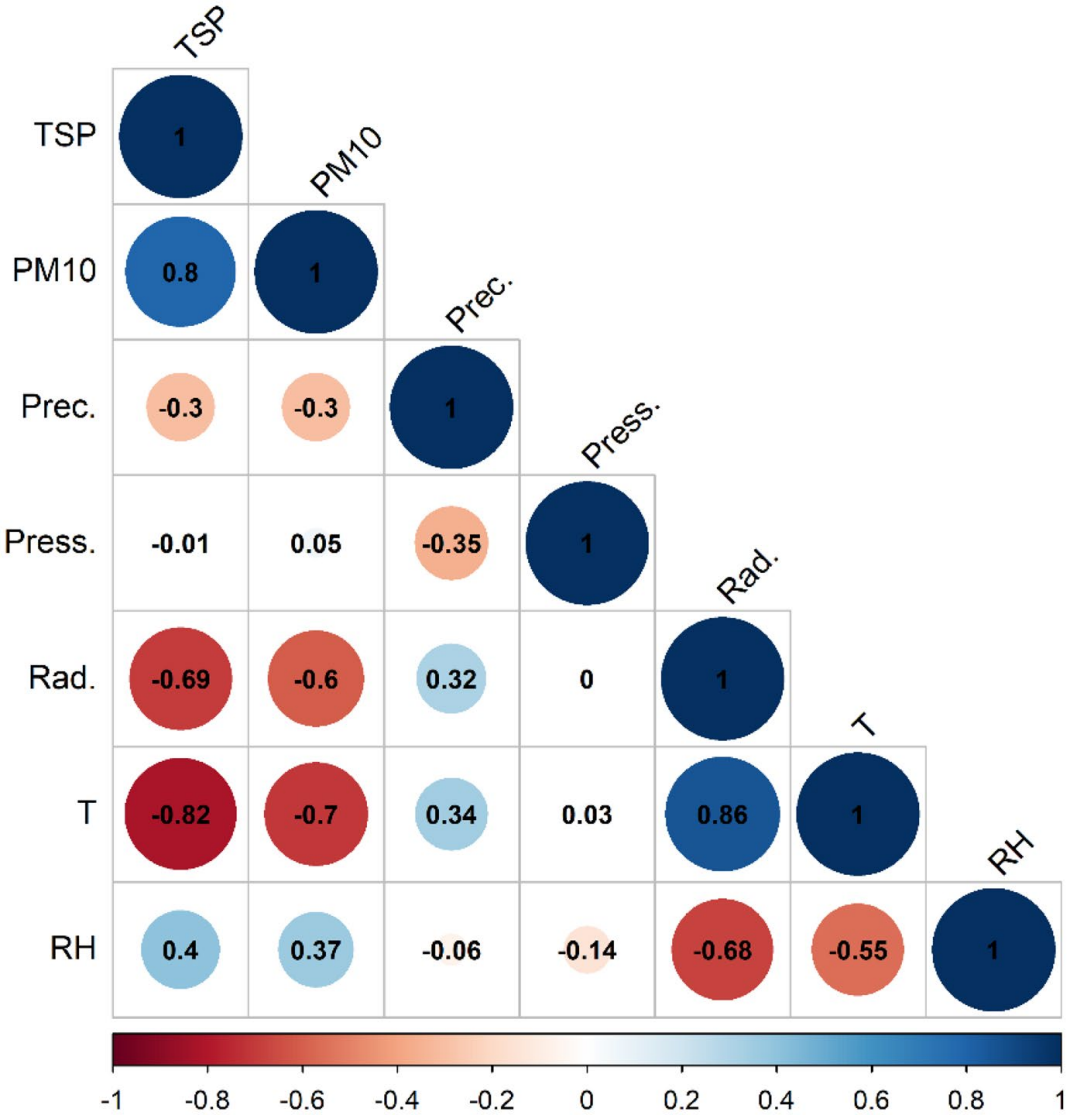
Spearman correlation coefficients between the studied parameters

Spearman correlation analysis was carried out for the TSP, PM_10_ and meteorological parameters. In total 114 monthly mean value was used, at a significance level of α = 0.05 the critical significant level was set to ± 0.2. As Fig. 10 shows, a very high positive correlation was found between TSP-PM_10_ (r = 0.8), and air temperature and solar radiation (r = 0.86). Moderate correlation was found between particulate matter and relative humidity (r = 0.37–0.4), and between precipitation quantity: air temperature and solar radiation (r = 0.32–0.34). A significant negative correlation was found between particulate matter and meteorological parameters: temperature (r = 0.7–0.82), solar radiation (0.6–0.69) and precipitation quantity (r = 0.3). Meanwhile, no correlation was found between air pressure variation particulate matter concentration.

## Conclusions

In this study temporal analysis was used to monitor and assess the overall variation characteristics of the concentration of PM_2.5_, PM_10_, total suspended particles (TSP) and meteorological parameters in the Ciuc basin Romania. The results revealed that the concentration of TSP, PM_10_ and PM_2.5_ were higher than the air quality index (AQI) limits set by WHO, especially during the cold period (winter).

On the other hand, according to our observations, significant differences were detected between seasons, namely in the cold period (November-March) the air pollution frequently exceeded the acceptable daily limits. Generally, the particulate matter concentration in the winter period is up to three orders of magnitude higher than in the summer period. Similar seasonal PM variations were reported from different regions of the world (Chen et al., 2013; Park, 2021). Besides the seasonal variation, the climatological and geographic parameters of the Ciuc depression are also responsible for these differences. Due to the closed type basin (surrounded by mountains) and frequent fog in the Ciuc basin, the accumulation of air pollutants in the winter has a number of negative effects on the exposed population (K. Bodor et al., 2021a, 2021b; Z. Bodor et al., 2020).

Based on our results, we can conclude that in order to reduce the particulate matter concentration in the Ciuc basin requires striker regulation, complex air pollution monitoring systems and clearer measures and action plans to reduce emissions as well. In order to achieve this, more renewable energy needs to be used rather than burning biomass.

The limitation of the present study was the PM_2,5_ data availability from 2017. Further research is needed to examine the trace elements content of particulate matter and also the differences between the pollution concentration in urban and suburban environments are required.

## Data Availability

The datasets generated during and/or analysed during the current study are available from the corresponding author on reasonable request.
